# Treatment-resistant depression and peripheral C-reactive protein

**DOI:** 10.1192/bjp.2018.66

**Published:** 2019-01

**Authors:** Samuel R. Chamberlain, Jonathan Cavanagh, Peter de Boer, Valeria Mondelli, Declan N.C. Jones, Wayne C. Drevets, Philip J. Cowen, Neil A. Harrison, Linda Pointon, Carmine M. Pariante, Edward T. Bullmore

**Affiliations:** 1 Department of Psychiatry, University of Cambridge, UK and Cambridgeshire and Peterborough NHS Foundation Trust, Cambridge, UK; 2 Institute of Health & Wellbeing, University of Glasgow, Glasgow, UK; 3 Neuroscience, Janssen Research & Development, Janssen Pharmaceutica NV, Beerse, Belgium; 4 The Maurice Wohl Clinical Neuroscience Institute, London, UK; 5a Neuroscience, Janssen Research & Development, London, UK; 5 Neuroscience, Janssen Research & Development, New Jersey, USA; 6 University of Oxford Department of Psychiatry, Warneford Hospital, Oxford, UK; 7 Brighton & Sussex Medical School, University of Sussex, Brighton, UK and Sussex Partnership NHS Foundation Trust, Swandean, UK; 8 Department of Psychiatry, University of Cambridge, UK; 10 Stress, Psychiatry and Immunology Laboratory & Perinatal Psychiatry, Maurice Wohl Clinical Neuroscience Institute, Kings College London, UK; 11 Immuno-Psychiatry, Immuno-Inflammation Therapeutic Area Unit, GlaxoSmithKline R&D, Stevenage, UK, Cambridgeshire and Peterborough NHS Foundation Trust, Cambridge, UK and Department of Psychiatry, University of Cambridge, UK

## Abstract

**Background:**

C-reactive protein (CRP) is a candidate biomarker for major depressive disorder (MDD), but it is unclear how peripheral CRP levels relate to the heterogeneous clinical phenotypes of the disorder.

**Aim:**

To explore CRP in MDD and its phenotypic associations.

**Method:**

We recruited 102 treatment-resistant patients with MDD currently experiencing depression, 48 treatment-responsive patients with MDD not currently experiencing depression, 48 patients with depression who were not receiving medication and 54 healthy volunteers. High-sensitivity CRP in peripheral venous blood, body mass index (BMI) and questionnaire assessments of depression, anxiety and childhood trauma were measured. Group differences in CRP were estimated, and partial least squares (PLS) analysis explored the relationships between CRP and specific clinical phenotypes.

**Results:**

Compared with healthy volunteers, BMI-corrected CRP was significantly elevated in the treatment-resistant group (*P* = 0.007; Cohen's *d* = 0.47); but not significantly so in the treatment-responsive (*d* = 0.29) and untreated (*d* = 0.18) groups. PLS yielded an optimal two-factor solution that accounted for 34.7% of variation in clinical measures and for 36.0% of variation in CRP. Clinical phenotypes most strongly associated with CRP and heavily weighted on the first PLS component were vegetative depressive symptoms, BMI, state anxiety and feeling unloved as a child or wishing for a different childhood.

**Conclusions:**

CRP was elevated in patients with MDD, and more so in treatment-resistant patients. Other phenotypes associated with elevated CRP included childhood adversity and specific depressive and anxious symptoms. We suggest that patients with MDD stratified for proinflammatory biomarkers, like CRP, have a distinctive clinical profile that might be responsive to second-line treatment with anti-inflammatory drugs.

**Declaration of interest:**

S.R.C. consults for Cambridge Cognition and Shire; and his input in this project was funded by a Wellcome Trust Clinical Fellowship (110049/Z/15/Z). E.T.B. is employed half time by the University of Cambridge and half time by GlaxoSmithKline; he holds stock in GlaxoSmithKline. In the past 3 years, P.J.C. has served on an advisory board for Lundbeck. N.A.H. consults for GlaxoSmithKline. P.d.B., D.N.C.J. and W.C.D. are employees of Janssen Research & Development, LLC., of Johnson & Johnson, and hold stock in Johnson & Johnson. The other authors report no financial disclosures or potential conflicts of interest.

Immunological mechanisms are increasingly implicated in the pathogenesis of depressive symptoms.^1^^–^^3^ Activation of the peripheral immune system has been consistently associated with major depressive disorder (MDD).^4^^–^^8^ However, it has also been anticipated that not all patients with MDD will be peripherally inflamed to the same extent. A deeper understanding of how peripheral immune biomarkers relate to some of the dimensions of clinical heterogeneity encompassed by a diagnosis of MDD could be an important step towards mechanistically stratified treatment of depression in the future.^3^^,^^9^^,^^10^

C-reactive protein (CRP) is an acute-phase protein that is widely used in clinical practice and has also been measured in many prior studies of MDD.^8^ A high-sensitivity assay for CRP is well-validated and accessible. CRP synthesis is induced in the liver by proinflammatory cytokines – especially interleukin 6 (IL-6) – in response to infection, inflammation and tissue damage. In a meta-analysis of 20 case–control studies,^8^ CRP was moderately increased ‘on average’ (Cohen's *d* = 0.47) in patients with MDD. However, there was significant heterogeneity of effect size between studies that may be attributable to clinical heterogeneity, with higher CRP in severe depression (Cohen's *d* = 0.50) than in mild/moderate depression (Cohen's *d* = 0.37), as well as methodological differences between studies.^11^

We were motivated to test the hypothesis that the clinically defined subgroup of patients with treatment-resistant depression would have the most abnormally increased CRP. An association between treatment resistance to monoaminergic antidepressant drugs and increased CRP is hypothetically predictable on clinical and mechanistic grounds. Clinical studies indicate that proinflammatory cytokines that induce CRP synthesis are increased in treatment-resistant MDD.^12^^,^^13^ Mechanistic studies have shown that proinflammatory cytokines can reduce the extracellular availability of serotonin by biasing expression of genes related to serotonin transport and tryptophan metabolism.^14^^,^^15^ Single studies have also reported that elevated CRP may be associated with other dimensions of clinical heterogeneity, namely atypical depression,^16^ childhood adversity,^17^ higher numbers of previous depressive episodes^18^ or anxiety in male patients.^19^

We measured CRP in four groups of participants: patients with MDD who are currently experiencing depression but are not receiving medication (untreated), patients who are currently depressed and are receiving medication (treatment-resistant), patients who are currently receiving medication but are not depressed (treatment-responsive) and healthy volunteers with no history of MDD or monoaminergic drug treatment. The primary hypothesis, that CRP would be most clearly increased above normal levels in treatment-resistant patients with MDD, was tested by planned analyses of between-group differences in mean CRP. In a secondary analysis, we took a more exploratory approach to the question of what other dimensions of clinical heterogeneity in the sample might be related to variation in CRP. We used the multivariate technique of partial least squares (PLS) to explore the relationships between CRP and multiple (139) clinical phenotypes – ranging from body mass index (BMI) to questionnaire items for depressive symptoms, anxiety states or history of childhood adversity.^20^^–^^23^ In this way, we could identify a subset of clinical phenotypes weighted strongly on latent dimensions of clinical heterogeneity that were predictive of higher CRP levels. We also tested the confirmatory hypothesis that scores on these clinical dimensions of peripheral inflammation would be higher in the subgroup of patients with treatment resistance defined *a priori*.

## Method

This was a non-interventional study, conducted as part of the Wellcome Trust Consortium for Neuroimmunology of Mood Disorders and Alzheimer's disease (NIMA). There were five clinical study centres in the UK: Brighton, Cambridge, Glasgow, King's College London and Oxford. All procedures were approved by an independent research ethics committee (National Research Ethics Service East of England, Cambridge Central, UK; approval number 15/EE/0092) and the study was conducted according to the Declaration of Helsinki. All participants provided informed consent in writing and received £100 compensation for taking part.

### Sample and eligibility criteria

We recruited four groups of participants, those with treatment-resistant depression, treatment-responsive depression, untreated depression and healthy volunteers.

For all participants, the following inclusion criteria applied: age 25–50 years; able to give informed consent; able to fast for 8 h and abstain from strenuous exercise for 72 h prior to venous blood sampling; and fluent English. The following exclusion criteria applied: pregnancy or breast feeding, alcohol or substance use disorder in the preceding 12 months, participation in an investigational drug study within the preceding 12 months, lifetime history of any medical disorder or current use of any medication (e.g. statins, corticosteroids, antihistamines, anti-inflammatory medications) likely to compromise interpretation of CRP (see Supplementary Material, available at https://doi.org/10.1192/bjp.2018.66).

Adult patients meeting DSM-5 criteria for MDD^24^ were recruited from National Health Service mental health and primary care services and from the general population by purposive advertising. Lifetime histories of bipolar disorder or non-affective psychosis were additional exclusion criteria. Diagnosis of MDD and other psychiatric disorders was ascertained by the Structured Clinical Interview for DSM-5.^25^ Current depressive symptom severity was defined by total scores from the 17-item Hamilton Rating Scale for Depression (HAM-D),^26^ and lifetime antidepressant medication use was quantified by the Antidepressant Treatment Response Questionnaire (ATRQ).^27^ The ATRQ was completed by a member of the study team via an interview with each participant. This structured instrument records all medications received for at least 6 weeks for treatment of depression, for current and past depressive episodes. For each medication ever received, the percentage improvement experienced by the participant during the corresponding episode was documented (<25%, 25–49% improved, 50–75% improved or >75% improved). Treatment response was conservatively defined as >75% improvement in depressive symptoms, as recalled by the participant. The ATRQ provides definitions for the minimum dose for each medication to be considered an adequate treatment course.^27^

Patients were assigned to one of three subgroups or strata, per protocol: treatment-resistant (DEP+MED+) patients who had total HAM-D score > 13 and had been medicated with a monoaminergic drug at a therapeutic dose for at least 6 weeks; treatment-responsive (DEP−MED+) patients who had total HAM-D score  < 7 and had been medicated with a monoaminergic drug at a therapeutic dose for at least 6 weeks; and untreated (DEP+MED−) patients who had HAM-D score > 17 and had not been medicated with a monoaminergic drug for at least 6 weeks. Cut-offs were defined *a priori* based on the literature. Total HAM-D score >17 is a standard threshold for entry into placebo-controlled treatment trials of MDD, whereas a lower threshold of total HAM-D score > 13 is typically used to define treatment-resistant depression, because there is usually some modest symptomatic response to treatment even if patients remain depressed.^28^^,^^29^

A group of healthy volunteers was recruited by advertising with no current or past history of any major psychiatric disorder as defined by DSM-5, and no history of monoaminergic drug treatment for any indication. Healthy volunteers completed the same screening and baseline assessments as patient groups (see below).

Age, gender, medical history, smoking status and family history were documented by semi-structured clinical interviews. Height and weight were measured for calculation of BMI (kg/m^2^).

### Questionnaire assessments

Psychological symptoms and childhood adversity were assessed by administration of the following questionnaires (see Supplementary Material): the Beck Depression Inventory (BDI v2.0^30^), the Spielberger State-Trait Anxiety Rating scale (STAI^31^), the Chalder Fatigue Score (CFS^32^), the Snaith-Hamilton Pleasure Scale (SHAPS^33^) and the Childhood Trauma Questionnaire (CTQ^34^).

### High-sensitivity CRP measurement

CRP was measured as one of many immunological markers in a venous blood sample drawn from each participant. Here, we focus on CRP because this has established utility as an immune biomarker of depression, having been widely used in case–control and epidemiological studies, and thus informing our hypothesis that CRP would be increased specifically in treatment-resistant depression. Participants fasted for 8 h and abstained from strenuous exercise for 72 h prior to venous blood sampling between 08:00 and 10:00. Patients taking psychotropic medication(s) continued their usual medication during the assessment day. High-sensitivity CRP was assayed via a central laboratory (see Supplementary Material).

### Statistical analysis

For analysis of between-group differences in high-sensitivity CRP and other variables, we first compared all participants with MDD to healthy volunteers, using planned *t*-tests. We then evaluated pairwise group differences with *post hoc t*-tests, provided the main effect of group was significant by one-way analysis of variance. When assumptions of normality were violated, data were appropriately transformed and/or non-parametric tests were used for inference. Cohen's *d* was reported for the effect size of high-sensitivity CRP corrected for BMI in each clinical group compared with healthy volunteers. Additionally, we compared the proportion of participants in each group who had clinically elevated CRP, defined as >3 mg/L.^35^^,^^36^ The threshold for statistical significance was defined as two-tailed *P* < 0.05 throughout.

To identify demographic and clinical phenotypes associated with variation in CRP across all study participants, we utilised the method of PLS, as implemented in JMP Pro software version 13.0.^37^ PLS is a multivariate technique for modelling relationships between a set of predictor (*X*) and response (*Y*) variables in terms of a set of mutually orthogonal latent factors, or PLS components.^21^^,^^22^^,^^38^^,^^39^ It requires no distributional assumptions and thus is robust against skewness. This same software was also used for other statistical tests and generation of violin plots for CRP across groups.

Here we modelled high-sensitivity CRP as the response variable, *Y*. The predictor variables, *X*, comprised gender, age, BMI, and education level as well as each of the 21 HAM-D, 11 CFS, 21 BDI, 28 CTQ, 40 STAI and 14 SHAPS questionnaire items. Data from all participants were included and missing data were imputed by sample means. Thus, the *Y* vector was (252 × 1) and the *X* matrix was (252 × 139). Because of the number of variables and the expectation that many variables would correlate with each other, other statistical approaches (such as linear regression) would not have been valid. In contrast, PLS is an ideal statistical technique under these circumstances.^21^^,^^22^^,^^38^^,^^39^ An initial PLS model was fitted including all predictor variables. We then used a two-step approach to identify the subset of predictor variables that significantly contributed to the model: first, we discarded individual *X* variables with low importance by conventional criteria (variable importance parameter < 0.8 and standardised absolute model coefficient less than the absolute magnitude of 0.05^40^); second, we utilised a more conservative approach of excluding variables whose standardised model coefficient had a 95% CI (constructed by bootstrapping the data 1000 times) that included zero. PLS models were fitted by leave-one-out cross-validation (non-linear iterative PLS (NIPALS) algorithm), and the optimal number of latent factors was selected by minimising the predictive residual sum of the squares. The statistical significance of the final model was confirmed by comparing the percentage of variation in *X* and *Y* accounted for in the experimental data compared with the null distributions of the percentage of *X* or *Y* variance sampled by bootstrapping (1000 iterations).

## Results

### Demographic and clinical data

The size of each group and their demographic and clinical characteristics are summarised in Table 1. The groups did not differ significantly in terms of demographic characteristics. As expected, *post hoc* tests indicated that each group differed significantly from each other group on HAM-D total score (least significant *t* = 4.19, d.f. = 248, *P* < 0.001). The mean number of failed pharmacological treatments for MDD episodes (<75% symptomatic response, defined by ATRQ) is listed for each clinical group in Table 1. The treatment-resistant group had more failed treatments than the untreated group (Wilcoxon *Z* = 2.843, *P* = 0.005); both the treatment-resistant group and the untreated group had significantly more failed treatments than the treatment-responsive group (Wilcoxon *Z* = 5.794, *P* < 0.001 and Wilcoxon *Z* = 3.079, *P* = 0.002, respectively). The majority of treatment-resistant patients were taking selective serotonin reuptake inhibitors (see Table 1 footnote). Summary statistics for questionnaire-based measures and comorbidities are provided in supplementary Tables 1 and 2.

**Table 1:** Demographic, clinical and high-sensitivity CRP data

	Mean (95% CI)/*n* (%)				Group test	
	Healthy volunteers *n* = 54	Treatment-responsive MDD *n* = 48	Treatment-resistant MDD *n* = 102^a^	Untreated MDD *n* = 48	Statistic	*P* value
Age, years	34.2 (32.3–36.2)	35.9 (33.6–38.3)	36.5 (35.1–38.0)	35.1 (32.6–37.6)	*F* = 1.14	0.34
Gender, female	37 (68.5%)	32 (66.7%)	72 (70.6%)	31 (64.6%)	*L* = 0.61	0.89
Education level	3.7 (3.4–4.0)	3.4 (3.1–3.7)	3.3 (3.1–3.5)	3.3 (3.0–3.6)	*K* = 6.42	0.09
**Smoking status**						
Never smoked	35 (64.8%)	34 (70.8%)	63 (61.8%)	33 (68.8%)	*L* = 2.36	0.88
Current smoker	9 (16.7%)	6 (12.5%)	18 (17.7%)	5 (10.4%)		
Ex-smoker	10 (18.5%)	8 (16.7%)	21 (20.6%)	10 (20.8%)		
HAM-D total score	0.8 (0.5–1.1)	4.0 (3.2–4.8)	18.3 (17.5–19.0)	20.5 (19.6–21.4)	*F* = 615.0	<0.001
Number of failed antidepressant drug treatments (lifetime)	N/A	1.1 (0.7–1.6)	2.7 (2.4–3.0)	1.8 (1.2–2.3)	*K* = 38.0	<0.001
BMI, kg/m^2^	25.5 (23.9–27.1)	27.8 (26.2–29.5)	27.6 (26.5–28.7)	26.4 (24.7–28.0)	*K* = 4.85	0.18
CRP, mg/L	1.3 (0.9–1.6)	2.1 (1.5–2.8)	3.1 (2.1–4.2)	2.50 (1.3–3.7)	*K* = 10.72	0.01
CRP>3 mg/L	4 (7.4%)	11 (22.9%)	26 (25.5%)	14 (29.2%)	*L* = 10.50	0.015
Log_10_ CRP	−0.1 (−0.2 to 0.0)	0.1 (0.0–0.3)	0.2 (0.1–0.3)	0.0 (−0.1 to 0.2)	*F* = 3.57	0.015
BMI-corrected CRP	−0.1 (−0.3 to 0.0)	0.0 (−0.1 to 0.1)	0.1 (0.0–0.2)	0.0 (−0.2 to 0.0)	*F* = 2.67	0.048
Cohen's *d* for case–control difference in BMI-corrected CRP		0.29	0.47	0.18		
Estimated sample size (*n*; case and control) for 80% power to detect difference in BMI-corrected CRP		188	73	486		

BMI, body mass index; CRP, C-reactive protein; *F,* one-way analysis of variance; HAM-D, Hamilton Rating Scale for Depression; *K*, Kruskal–Wallis test; *L*, likelihood ratio; MDD, major depressive disorder.

a. The majority of treatment-resistant patients were taking selective serotonin reuptake inhibitors (70%) with smaller numbers exposed to noradrenergic and specific serotonergic reuptake inhibitors (15%), mixed reuptake inhibitors (25%), tricyclic antidepressants (4%), mood stabilisers (4%) and dopamine receptor antagonists (3%). Treatment-responsive patients were likewise predominantly treated with selective serotonin reuptake inhibitors (85%), followed by mixed reuptake inhibitors (25%), noradrenergic and specific serotonergic reuptake inhibitors (11%) or tricyclic antidepressants (4%). All treatment-resistant patients were taking at least one conventional antidepressant monoaminergic drug.

### CRP

Mean high-sensitivity CRP concentrations (and 95% CIs) are shown in Table 1. Mean CRP was significantly increased in all patients with MDD compared with healthy controls (Wilcoxon Z = 2.7, *P* = 0.007). Both treatment-resistant and treatment-responsive groups had significantly higher mean high-sensitivity CRP than controls (Wilcoxon *Z* = 2.9, *P* = 0.004 and Wilcoxon *Z* = 2.6, *P* = 0.010, respectively). Across all patients with MDD, drug treatment class did not significantly affect CRP levels (*F* = 0.799, *P* = 0.572), nor did the number of failed treatments in the past (*F* = 0.245, *P* = 0.621).

The proportion of participants with high-sensitivity CRP levels exceeding the conventional threshold value of 3 mg/L was also significantly different between the pooled MDD groups and controls (likelihood ratio χ^2^ = 10.01, *P* = 0.002). Treatment-resistant, untreated and treatment-responsive MDD groups all had significantly increased proportions of participants with high-sensitivity CRP > 3 mg/L compared with healthy volunteers (likelihood ratio χ^2^ = 8.4, *P* = 0.004; likelihood ratio χ^2^ = 8.6, *P* = 0.003; and likelihood ratio χ^2^ = 5.0, *P* = 0.025, respectively). No other *post hoc* test was statistically significant; that is, depressed groups did not differ significantly from each other (all *P* > 0.09).

### Log-transformed and BMI-corrected CRP

**Fig. 1:**
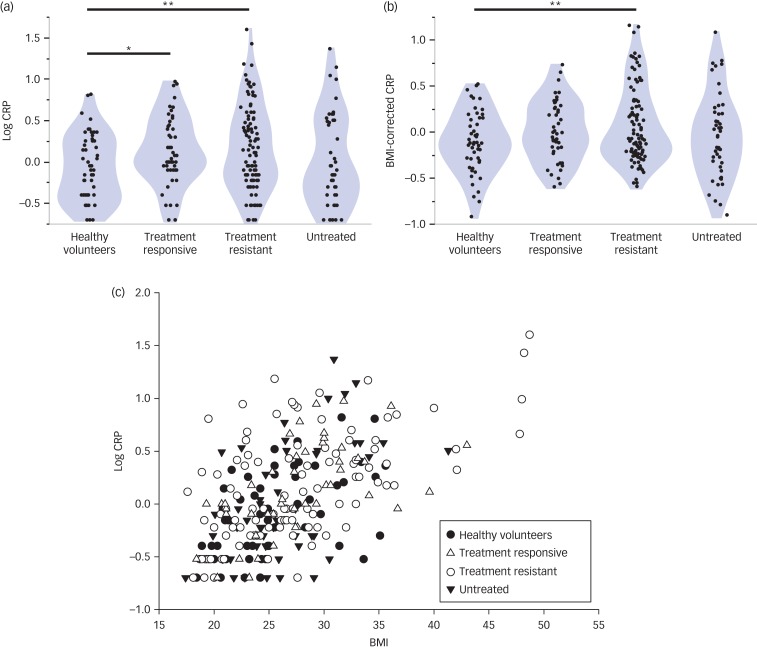
High-sensitivity CRP and its relationship with BMI.

The distributions of high-sensitivity CRP were positively skewed (moment skewness: 5.08)^41^ and therefore were normalised by base log_10_ transform (see Fig. 1). Log_10_ CRP was significantly increased in all patients with MDD compared with controls (*t* = 2.81, d.f. = 250, *P* = 0.004). Only treatment-resistant and treatment-responsive patients had significantly higher log_10_ CRP than controls (*t* = 3.07, d.f. = 248, *P* = 0.002 and *t* = 2.32, d.f. = 248, *P* = 0.021, respectively).

(a) Violin plots of log_10_ CRP for each of three subgroups of patients with MDD and healthy volunteers. (b) Violin plots of BMI-corrected CRP (log_10_ CRP regressed on log_10_ BMI) for each of three subgroups of patients with MDD and healthy volunteers. (c) Scatterplot of BMI versus log_10_ CRP (Spearman's rho = +0.57, *P* < 0.001), with points coded by sample group. The main effects of group were significant for (a) and (b) (one-way analysis of variance, *F* = 3.57, *P* = 0.015; *F* = 2.67, *P* = 0.048). *P < 0.05, **P < 0.01 significant pairwise difference between groups by *post hoc t*-tests. BMI, body mass index; CRP, C-reactive protein; MDD, major depressive disorder.

As anticipated by prior studies,^42^^–^^44^ there was a significant positive correlation between BMI and log_10_ CRP across all study participants (Spearman's rho = 0.56, d.f. = 250, *P* < 0.001; Fig. 1). Because BMI data were also positively skewed (moment skewness: 1.03),^41^ we regressed log_10_ CRP on log_10_ BMI and used the residuals as estimates of BMI-corrected CRP (Fig. 1). BMI-corrected CRP was significantly elevated in all patients with MDD compared with controls (*t* = 2.24, d.f. = 238, *P* = 0.026). *Post hoc t*-tests indicated that only the treatment-resistant patients had significantly higher mean BMI-corrected CRP than the controls (*t* = 2.71, d.f. = 236, *P* = 0.007; Cohen's *d* = 0.47).

To assess the possible confounding effect of symptom severity, we identified the subgroup of treatment-resistant patients (*n* = 48) that had a total HAM-D score > 17, thereby corresponding to the cut-off used to define the untreated group. We confirmed that BMI-corrected CRP was abnormally increased in treatment-resistant patients with HAM-D > 17 (*t* = 3.0, *P* = 0.004) with a case–control difference of similar size (Cohen's *d* = 0.43) to that of treatment-resistant patients with HAM-D > 13.

### PLS analysis of the relationship between CRP and clinical variables

**Fig. 2:**
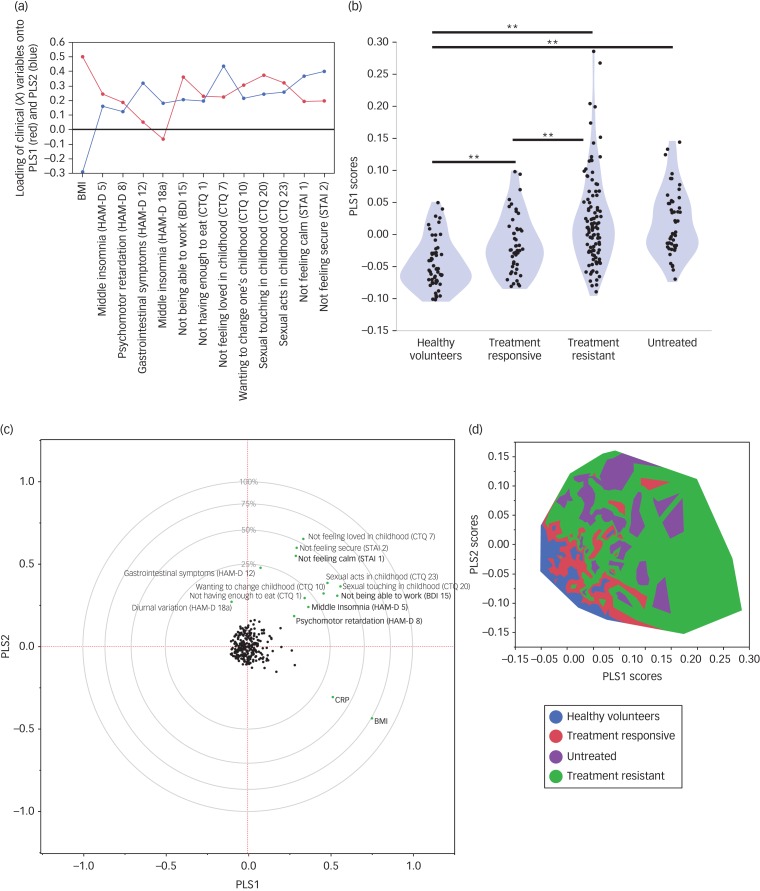
Partial least squares analysis of the relationships between high-sensitivity CRP and clinical phenotypes.

A total of 13 out of 139 clinical phenotypes passed criterion for an important effect on CRP levels. Iterative cross-validation of the PLS model including only these important variables yielded an optimal two-factor solution (Fig. 2 and Supplementary Fig. 2), which accounted in total for 34.7% of variation in clinical measures (*X*), and for 36.1% of variation in CRP (*Y*). This differed significantly from the proportions of variance expected under the null hypothesis (percentage variance (*X*) = 12.3%, 95% CI 12.1–12.5%; percentage variance (*Y*) = 2.7%, 95% CI 1.9–3.0%).

(a) Loadings of clinical (*X*) variables onto the first two PLS components. These 13 variables passed the first criterion for an important effect on CRP variance. (b) Violin plot of PLS1 *X* scores for each of three subgroups of patients with MDD and healthy volunteers. **P < 0.01 significant pairwise difference between groups by *post hoc t*-tests. (c) Plot of the clinical (*X*) and CRP (*Y*) variables in the space of the first two PLS components. The clinical variables named in bold font passed both criteria for an important effect on CRP; the variables named in normal font passed the first criterion but not the more conservative second criterion. (d) Contour plot for distribution of study participants in the space of the first two PLS components, color-coded by group, confirming that patients with treatment-resistant depression had high scores on the clinical syndrome of variables represented by PLS1. BDI, Beck Depression Inventory; CRP, C-reactive protein; CTQ, Childhood Trauma Questionnaire; HAM-D, Hamilton Rating Scale for Depression; MDD, major depressive disorder; PLS, partial least squares; PLS1, partial least squares component 1; PLS2, partial least squares component 2; STAI, Spielberger State-Trait Anxiety Rating scale.

The first PLS component (PLS1) accounted for 26.7% of the variation in high-sensitivity CRP. Positive scores on PLS1 indicated higher CRP. The clinical phenotypes that were significantly weighted on PLS1 were higher BMI, not feeling loved in childhood (CTQ item seven), not feeling calm (STAI item one), wanting to change one's family in childhood (CTQ item ten), psychomotor retardation (HAM-D item eight), middle insomnia (HAM-D item five) and not being able to work (BDI item 15). The PLS1 scores for individual participants differed significantly between groups (*F* = 19.88, d.f. = 3248, *P* < 0.001; Fig. 2). PLS1 scores were highest in the treatment-resistant group, followed by the untreated group, the treatment-responsive group and the healthy volunteers.

The second PLS component (PLS2) orthogonally explained 9.4% of variation in CRP. PLS scores in each group, and variables loading significantly onto PLS2, are summarised in Supplementary Fig. 3. Positive scores on PLS 2 indicated lower CRP. PLS2 scores differed significantly between groups (*F* = 24.34, d.f. = 3248, *P* < 0.001). Untreated patients had the highest PLS2 scores, followed by treatment-resistant patients, treatment-responsive patients and healthy volunteers.

## Discussion

This is the first study to measure peripheral CRP with the same high-sensitivity assay across a large sample of patients with MDD (*n* = 198) prospectively stratified in terms of their current and past history of treatment with monoaminergic antidepressant drugs. We replicated the well-established finding that CRP is significantly increased ‘on average’ in patients with MDD, screened for physical comorbidity, and compared with healthy volunteers who did not differ in terms of age, gender, BMI and cigarette smoking status. However, we also found some evidence for our primary hypothesis that CRP was most increased in the subgroup of patients with treatment-resistant depression (*n* = 102). The standardised size of the case–control difference in CRP between healthy volunteers and treatment-resistant patients (Cohen's *d* = 0.47) was higher than the case–control difference for treatment-responsive (0.29) or untreated patients (0.18). Controlling for non-normality of the CRP distribution, and for the strong positive correlation between CRP and obesity, we found that the case–control difference in CRP remained significant only for the subgroup of treatment-resistant patients. These results of planned analysis are consistent with the hypothesis that peripheral inflammation is a marker or risk factor for treatment-resistant depression. It should be noted that CRP was somewhat elevated even in treatment-responsive patients, suggesting that a degree of elevated CRP could be trait-related rather than related to current symptoms or treatment status.

Taking a convergent but more exploratory approach to the data, we used multivariate analysis to identify two dimensions of clinical heterogeneity that were predictive of CRP. We found that a subset of clinically measured phenotypes explained approximately 36% of the variance in CRP. High BMI, high scores on vegetative symptoms of depression, low scores on calmness and a history of childhood adversity were all predictive of increased CRP. As expected from the results of our primary analysis, we confirmed that the group of patients defined *a priori* in terms of treatment resistance had the highest scores on this clinical profile associated with high-sensitivity CRP.

### Treatment-resistant depression and peripheral inflammation

Monoamine reuptake inhibitors and related drugs are evidence-based pharmacological treatments for MDD, but response failure afflicts approximately 30% of patients.^45^^,^^46^ Because of the global burden of disability attributable to MDD,^47^ the search for improved understanding of biomarkers of therapeutic resistance to current first-line treatment options is pressing.^1^^,^^48^ Our results provide fresh evidence that patients with treatment-resistant depression have the most abnormally increased CRP levels compared with both treatment-responsive and currently untreated patients. To our knowledge, a specific relationship between CRP and monoaminergic antidepressant drug treatment resistance has not been demonstrated previously, although there is evidence both for increased proinflammatory cytokine concentrations^3^^,^^14^^,^^49^ and for increased peripheral expression of cytokine related genes^12^ in treatment-resistant depression. There is also some evidence that baseline inflammatory markers may be useful predictors of treatment response in MDD.^50^^,^^51^ In a rat model of treatment-resistant depression, elevated CRP at baseline differentiated responders from non-responders to ketamine, an *N*-methyl-D-aspartate receptor antagonist with anti-inflammatory and antidepressant effects.^52^ At a cellular level, neurons, microglia and macrophages respond to inflammatory challenges by activating metabolic pathways that reduce the synaptic availability of serotonin and catalyse the conversion of tryptophan to kynurenine and its putatively neurotoxic, glutamatergic agonist metabolites.^15^^,^^53^^–^^55^ These effects of inflammation on serotonin transport and tryptophan metabolism may constitute a mechanism by which peripheral inflammation is associated with lack of therapeutic response to selective serotonin reuptake inhibitors.^56^

### Clinical phenotypes predictive of increased CRP in depression

Obesity and its cardiovascular sequelae have been repeatedly associated with increased CRP.^43^^,^^57^^,^^58^ In this study, which excluded patients with a lifetime history of medical disorders including atherosclerosis and diabetes, we confirmed that higher BMI was strongly associated with higher CRP levels. One mechanistic explanation is that macrophages constitute up to 60% of cells in adipose tissue and can release large amounts of IL-6, which is a key driver of CRP synthesis.^59^ Therefore, it is not surprising that inflammation (CRP) and obesity (BMI) were related herein; however, we do not consider that this association trivially accounts for increased CRP in treatment-resistant depression. The groups did not differ significantly in baseline BMI and the case–control difference remained significant for the treatment-resistant patients even after statistical regression to control for individual differences in BMI.

Of all the depressive symptoms measured, so-called vegetative symptoms (psychomotor retardation, insomnia, difficulty getting started, difficulty working) were more important in explaining higher CRP. These findings are consistent with prior reports that somatic but not cognitive symptoms of depression were associated with increased CRP.^60^ Vegetative symptoms of depression are akin to the illness or sickness behaviour that has been repeatedly demonstrated in animal models and experimental medicine studies of humans exposed to acute proinflammatory challenge.^2^^,^^61^ We also found evidence that state anxiety was related with CRP, which is compatible with prior data linking acute endotoxin exposure to anxious and depressive states in healthy volunteers.^62^

It is established that childhood trauma increases risk of later mental health disorders, including depression.^63^ In a meta-analysis, individuals exposed to childhood trauma had significantly elevated levels of CRP in adulthood, albeit with a small effect size (Fisher's *Z* = 0.10).^64^ In a longitudinal study of female adolescents at risk of depression, childhood adversity was found to promote subsequent clustering of depression and inflammation.^65^ These results are compatible with our findings that feeling unloved in childhood and wanting to change one's family in childhood were significantly correlated with higher CRP in adults.

### Methodological issues

Because of the case–control design, between-group differences in CRP could theoretically be confounded by other factors influencing peripheral inflammation. However, we excluded patients with inflammatory disorders or anti-inflammatory drug treatment, and the groups did not differ on demographic characteristics. The lack of statistically significant case–control differences in BMI-corrected CRP for the comparisons between healthy volunteers and the treatment-responsive and untreated MDD groups could theoretically reflect the smaller sizes of these groups compared with the treatment-resistant MDD group. However, power calculations indicated that the case–control differences in BMI-corrected CRP would probably not have been significant even if the treatment-responsive group had the same size as the resistant group (Table 1). Although the untreated MDD group had not received antidepressant treatment for at least six weeks, the majority (*n* = 28, 58.3%) had received at least one such treatment in the past (the average number of historically failed treatments in this group was 1.8). As such, this group had some degree of heterogeneity, including both treatment-naïve individuals and those who would have been expected to be treatment-resistant if currently medicated. Treatment resistance was defined by inadequate response to the current drug treatment whereas some other criteria for treatment resistance stipulate a failed response to at least two drugs of different mechanisms of action. There are multiple definitions of treatment resistance used in the field; our choice is widely used. The study was not planned or powered to test differences in CRP between subgroups defined by dose or type of current antidepressant medication. We did not find that CRP differed significantly as a function of antidepressant medication class, nor as a function of total number of previous failed treatments (as measured by the ATRQ). A limitation in assessing prior medications was the use of retrospective self-report, albeit based on a comprehensive structured instrument completed by an interviewer. The sample was recruited from the UK population, which is known to differ from the USA and other populations in terms of BMI and other factors that can influence the numerical distribution of CRP, and this may mitigate generalisability of our results, as would our exclusion of people with inflammatory disorders. Finally, CRP is only one of many markers of peripheral inflammation that have been or could be linked to depression. Although these data demonstrate that CRP is robustly associated with treatment-resistant depression, we do not claim that CRP is necessarily the best of all possible peripheral blood biomarkers of treatment-resistant depression.

### Conclusions

MDD is associated with increased CRP compared with healthy volunteers and the case–control difference appears higher in treatment-resistant depression. Increased CRP and treatment resistance were also associated with other aspects of clinical heterogeneity in depression including obesity, vegetative symptoms of fatigue and sleep disturbance, state anxiety and a history of childhood adversity. We suggest there may be a clinically and immunologically diagnosable subsyndrome of ‘inflamed depression’ comprising the patients with MDD most likely to benefit therapeutically from second-line treatment with anti-inflammatory drugs.^36^

## Supplementary material

For supplementary material accompanying this paper visit https://doi.org/10.1192/bjp.2018.66. click here to view supplementary material
