# Supplementary material for: Treatment-resistant depression and peripheral C-reactive protein
Source: Br J Psychiatry. 2019 Jan;214(1):11–9. doi: 10.1192/bjp.2018.66 (PMC6124647; doi:10.1192/bjp.2018.66)
Supplement: Supplementary file 1 [file S0007125018000661sup001.zip › Supplementary material 23.1.18.docx]

**Treatment-resistant depression and peripheral C-reactive protein**

Samuel R. Chamberlain^1,2^*, Jonathan Cavanagh^3^, Peter de Boer^4^, Valeria Mondelli^5^, Declan Jones^6^, Wayne C. Drevets^6^, Philip J. Cowen^7^, Neil A. Harrison^8,9^, Linda Pointon^1^, NIMA Consortium, Carmine M. Pariante^10#^, Edward T. Bullmore^1,2,11#^

^1^ Department of Psychiatry, School of Clinical Medicine, University of Cambridge, CB2 0SZ, UK

^2^ Cambridgeshire and Peterborough NHS Foundation Trust, Cambridge, CB21 5EF, UK

^3^ Sackler Centre, Institute of Health & Wellbeing, University of Glasgow, Sir Graeme Davies Building , Glasgow, G12 8TA, UK

^4^ Neuroscience, Janssen Research & Development, Janssen Pharmaceutica NV, Turnhoutseweg 30, B-2340, Beerse, Belgium

^5^ The Maurice Wohl Clinical Neuroscience Institute, Cutcombe Road, London, SE5 9RT, UK

^6^ Neuroscience, Janssen Research & Development, LLC, Titusville, NJ, 08560, USA

^7^ University Department of Psychiatry, Warneford Hospital, Oxford, OX3 7JX, UK

^8^ Brighton & Sussex Medical School, University of Sussex, Brighton, BN1 9RR, UK

^9^ Sussex Partnership NHS Foundation Trust, Swandean, BN13 3EP, UK

^10^ Stress, Psychiatry and Immunology Lab & Perinatal Psychiatry, Maurice Wohl Clinical Neuroscience Institute, Kings College, London, SE5 9RT, UK

^11^ Immuno-Psychiatry, Immuno-Inflammation Therapeutic Area Unit, GlaxoSmithKline R&D, Stevenage SG1 2NY, UK

**Detailed participant eligibility criteria**

**Inclusion Criteria**

*Participants with MDD*

· Major depressive disorder diagnosed by structured clinical interview in accordance with DSM

criteria

· HAM-D score at baseline

a. Treatment resistant subgroup > 13

b. Untreated subgroup > 17

c. Treatment responsive subgroup < 7

*All Participants (including Healthy Volunteers)*

· Aged 25-50 years inclusive

· Able and willing to give informed consent, including consent to sharing of clinical information

with the participant’s general practitioner

· Able and willing to fast for 8 hours prior to blood draw

· Willing to abstain from strenuous exercise for 72 hours prior to assessment

· Able to speak, write and understand English

**Exclusion Criteria**

*Participants with MDD*

· Life time history of bipolar disorder or non-affective psychosis

Healthy Participants

· Personal history of MDD or treatment with a monoaminergic antidepressant for depressive

symptoms or any other indication

· Current or past history of any major psychiatric disorder as defined by DSM-V

*All Participants*

· Concurrent medication likely to compromise the interpretation of immunological data (including,

but not limited to, corticosteroids or any other substance to be determined by the Principal

Investigator or delegate)

· Pregnancy or breast feeding

· Active alcohol or drug abuse or dependence in the last 6 months

· Participation in clinical trial of an investigational drug within the last 12 months

· Lifetime history of any serious medical disorder likely to compromise the interpretation of

immunological data (including, but not limited to, immunological disorders, cardiovascular

disorders, malignancies or infection, or any other condition to be determined by the Principal

Investigator or delegate)

**Details of assessments**

Age, gender, medical history, and family history were documented using semi-structured clinical interview. Record was made of height and weight, for the purposes of calculating body mass indices (BMI). Presence of MDD and other mental disorders was recorded using the validated Structured Clinical Interview (DSM-5 version) ([Spitzer *et al*, 1996](#_ENREF_9)), and antidepressant history was documented using the Antidepressant Treatment Response Questionnaire (ATRQ) ([Desseilles *et al*, 2011](#_ENREF_4)). Our assessment then focused on quantifying the nature and degree of depressive symptoms, state and trait anxiety, fatigue, ability to experience pleasure, and history of childhood trauma, using the following validated instruments:

17-item Hamilton Rating Scale for Depression (HAM-D) ([Hamilton, 1960](#_ENREF_5)). The HAM-D is a clinician-administered rating scale designed to assess the severity of symptoms in participants diagnosed with depression with a score range of 0 to 52. It is the most widely used symptom severity measure for depression. The HAM-D has an inter-rater reliability correlation of r = .90 and the internal consistency of the measure is reported to be high with a coefficient alpha of 0.88. Criterion related validity for this measure is high ([Knesevich *et al*, 1977](#_ENREF_6)).

Beck Depression Inventory (BDI) version 2 ([Beck and Beamesderfer, 1974](#_ENREF_1)). A 21 item self-report questionnaire, with each answer being scored on a scale value of 0 to 3. Cutoffs have been standardized to indicate minimal depression, 0–13: mild depression, 14-19; moderate depression, 20- 28; and severe depression 29–63. As a self-report measure, it has been validated against clinical interviews by trained clinicians. The BDI is positively correlated with the Hamilton Depression Rating Scale with a Pearson r of 0.71, and has a high one-week test–retest reliability (Pearson r =0.93). The test also has high internal consistency (α=.91).

Spielberger State-Trait Anxiety Rating Scale (STAI) ([Spielberger *et al*, 1983](#_ENREF_8)). The STAI is a commonly used self-report measure of trait and state anxiety. The instrument has 20 items for assessing trait anxiety and 20 for state anxiety. State anxiety items include: “I am tense; I am worried” and “I feel calm; I feel secure.” Trait anxiety items include: “I worry too much over something that really doesn’t matter” and “I am content; I am a steady person.” All items are rated on a 4-point scale (from “Almost Never” to “Almost Always”). Higher scores indicate greater anxiety. Internal consistency coefficients for the scale have ranged from .86 to .95; test-retest reliability coefficients have ranged from .65 to .75 over a 2-month interval ([Spielberger *et al*, 1983](#_ENREF_8))

Chalder Fatigue Scale ([Chalder *et al*, 1993](#_ENREF_3)). The Chalder Fatigue Scale (CFS) is a 14-item self-report instrument to measure the severity of fatigue in adults. Physical (e.g. “Do you need to rest more?”) and mental symptoms (e.g. “Do you have problems thinking clearly?”) are evaluated. Each item is scored on a 4-point scale ranging from “better than usual” to “much worse than usual”. The internal consistency range from .88 to .90 and the validity is .85

Snaith-Hamilton Pleasure Scale (SHAPS) ([Snaith *et al*, 1995](#_ENREF_7)). Anhedonia, the inability to experience pleasure, is a common feature of depression. The SHAPS is a short, 14-item instrument to measure anhedonia, which has been shown to be valid and reliable in normative and clinical samples ([Snaith *et al*, 1995](#_ENREF_7)). Each of the 14 items has a set of four response categories: Definitely Agree (= 1), Agree (= 2), Disagree (= 3), and Definitely Disagree (= 4). A higher total score indicates higher levels of current anhedonia.

Childhood Trauma Questionnaire (CTQ) ([Bernstein *et al*, 1994](#_ENREF_2)). The CTQ is a standardized, retrospective 28-item self-report inventory that measures the severity of different types of childhood trauma, producing five clinical subscales each comprised of five items: Emotional Abuse, Physical Abuse, Sexual Abuse, Emotional Neglect, Physical Neglect. The measure also includes a three-item Minimization/Denial scale indicating the potential underreporting of maltreatment. Participants respond to each item in the context of “when you were growing up” and answer according to a five point Likert scale ranging from “never” = 1 to “very often” = 5, producing scores of 5 to 25 for each trauma subscale. The three items comprising the Minimization/Denial scale are dichotomized (“never” = 0, all other responses = 1) and summed; a total of one (1) or greater “suggests the possible underreporting of maltreatment (false negatives)”.

**CRP assay**

Blood samples were allowed to coagulate for 30-60 minutes then centrifuged at 1600 Relative Centrifugal Force (RCF) for 15 mins. 1ml of the resultant sample was then transferred to a white-topped serum tube using a pipette, and transported at room temperature to a central laboratory (Q^2^ solutions). Samples were exposed to anti-CRP-antibodies on latex particles, and the increase in light absorption due to complex formation was used to quantify CRP levels, using Turbidimetry on Beckman Coulter AU analyzers. Pentameric CRP was analysed on the day of receipt rather than in batches, and the coefficients of variation for three concentrations of CRP are shown below:

| **n = 80** | **Within-run (Intra)** | | **Total (Inter)** | |
| --- | --- | --- | --- | --- |
| **Mean mg/L** | **SD** | **CV%** | **SD** | **CV%** |
| 10.9 | 0.31 | 2.81 | 0.65 | 5.99 |
| 89.56 | 2.24 | 2.49 | 2.29 | 2.55 |
| 168.07 | 1.71 | 1.02 | 2.17 | 1.29 |

For three participants, the CRP result was outside the laboratory reference range, and there was evidence of minor infection in the preceding 2-weeks. For these subjects, CRP measurement was repeated after recovery from infection and the second measurement was used subsequently.

**Supplementary Analysis 1: group-level difference in CRP using identical HAM-D thresholds for treatment-resistant and untreated cases**

Per Protocol, and as reported in the Manuscript, a threshold of HAM-D > 13 was used to define treatment-resistant depression; and HAMD > 17 for untreated cases.

HAM-D total scores did not correlate significantly with CRP or BMI-corrected CRP (Spearman’s r, both P > 0.15). Nonetheless, to rule out potential impact of this differential criterion on group-level CRP differences, secondary analysis was conducted by excluding treatment-resistant cases with HAM-D > 17. This rendered the same operational definition for treatment-resistant and un-treated cases by HAM-D 17 item total scores.

Forty-eight treatment-resistant cases had HAM-D < 18, and these participants’ data were excluded in the secondary analysis. As expected, HAM-D total scores did not differ significantly between treatment-resistant and untreated MDD groups (t = 1.0, P = 0.30). CRP was significantly elevated in MDD cohorts viewed collectively, compared to healthy volunteers (Wilcoxon Z = 2.75, P = 0.006). Both treatment-resistant and untreated MDD groups had significantly higher hs-CRP than healthy volunteers (Wilcoxon Z = 3.2, P = 0.001 and Wilcoxon Z = 2.6, P = 0.010, respectively). Treatment-resistant MDD cases had significantly higher CRP than untreated cases (Wilcoxon Z = 2.0, p = 0.042). The main effect of group was significant (Wilcoxon Chi-Square = 12.66, df = 3, P = 0.005). No other post-hoc tests were statistically significant.

BMI-corrected CRP was significantly elevated in MDD cohorts viewed collectively, compared to healthy volunteers (t = 2.20, df = 193, P = 0.029). Post-hoc tests indicated that the treatment-resistant group had significantly higher BMI-corrected CRP than healthy volunteers (t = 3.0, P = 0.004; Cohen’s D = 0.43). The treatment-resistant cases also had significantly higher BMI-corrected CRP than untreated cases (t = 2.0, P = 0.045; Cohen’s D = 0.29). The main effect of group was significant (F = 3.1, df = 3,191, P = 0.03). BMI-corrected CRP did not differ significantly from healthy volunteers for treatment-responsive MDD group (t = 1.48, df = 191, P = 0.14; Cohen’s D = 0.21), nor for the untreated MDD group (t = 0.88, df = 191, P = 0.379; Cohen’s D = 0.13). No other post-hoc tests were statistically significant (all P > 0.07).

**Table S1. Summary statistics for all questionnaire-based measures**

|  | **Mean (Standard Deviation)** | | | | **Welch Test** | |
| --- | --- | --- | --- | --- | --- | --- |
|  | **Healthy Volunteers^a^** | **Treatment-Responsive^b^** | **Treatment Resistant^c^** | **Untreated^d^** | **W** | **p** |
| **BDI total score** | 1.4 (1.7)**^b,c,d^** | 9.9 (9.1)**^a,c,d^** | 27.0 (10.1)**^a,b,d^** | 23.7 (8.7)**^a,b,c^** | 286.562 | <0.001 |
| **Spielberger State-Trait Anxiety Rating Scale, State** | 26.4 (5.9)**^b,c,d^** | 36.0 (10.1)**^a,c,d^** | 52.4 (12.5)**^a,b^** | 52.8 (10.9)**^a,b^** | 134.187 | <0.001 |
| **Spielberger State-Trait Anxiety Rating Scale, Trait** | 27.3 (4.8)**^b,c,d^** | 43.7 (10.8)**^a,c,d^** | 62.7 (9.2)**^a,b^** | 60.5 (9.4)**^a,b^** | 278.967 | <0.001 |
| **Chalder Fatigue Scale** | 10.5 (3.3)**^b,c,d^** | 14.3 (5.3)**^a,c,d^** | 21.1 (5.5)**^a,b^** | 19.7 (5.3)**^a,b^** | 83.439 | <0.001 |
| **Snaith-Hamilton Pleasure Scale** | 16.2 (3.6)**^b,c,d^** | 22.5 (7.0)**^a,c,d^** | 32.5 (7.1)**^a,b^** | 31.6 (5.8)**^a,b^** | 153.654 | <0.001 |
| **CTQ, Emotional Abuse** | 6.6 (2.7)**^b,c,d^** | 9.6 (4.7)**^a,c,d^** | 12.3 (5.6)**^a,b^** | 12.5 (5.1)**^a,b^** | 31.851 | <0.001 |
| **CTQ, Physical Abuse** | 5.8 (2.9)**^c^** | 6.8 (3.6) | 7.5 (4.3)**^a^** | 7.0 (3.2) | 2.907 | 0.038 |
| **CTQ, Sexual Abuse** | 5.6 (2.4)**^c,d^** | 6.6 (4.2) | 7.4 (5.2)**^a^** | 7.5 (5.4)**^a^** | 3.492 | 0.018 |
| **CTQ Emotional Neglect** | 8.5 (3.8)**^b,c,d^** | 11.8 (4.9)**^a,c,d^** | 14.2 (5.3)**^a,b^** | 14.4 (5.7)**^a,b^** | 22.344 | <0.001 |
| **CTQ, Physical Neglect** | 6.1 (1.7)**^b,c,d^** | 7.5 (3.2)**^a^** | 8.4 (3.6)**^a^** | 8.6 (3.1)**^a^** | 13.420 | <0.001 |

Superscript letters indicate that the group differs significantly from the other group by post hoc Least Significant Difference tests at *P*<0.05. Welch tests were used for composite tests due to heterogeneity of variance across groups.

**Table S2. Comorbid non-psychotic psychiatric disorders in depressed patient groups**

|  | **N [%] of cases in group with disorder** | | | | **Likelihood Ratio** | |
| --- | --- | --- | --- | --- | --- | --- |
|  |  | **Treatment- Responsive^b^** | **Treatment Resistant^c^** | **Untreated^d^** | **L** | **p** |
| **Panic Disorder** |  | 2 [4.2%] | 13 [13.1%] | 3 [6.4%] | 3.927 | 0.140 |
| **Social phobia** |  | 1 [2.1%] | 12 [12.1%] | 3 [6.5%] | 5.289 | 0.071 |
| **Specific phobia** |  | 0 [0%] | 2 [1%] | 0 [0%] | 2.710 | 0.258 |
| **Obsessive Compulsive Disorder** |  | 1 [2.1%] | 10 [10.2%] | 2 [4.3%] | 4.340 | 0.114 |
| **Post-traumatic stress disorder** |  | 2 [4.2%] | 12 [12.4%]^d^ | 1 [5.7%]^c^ | 6.248 | 0.044 |
| **Generalized Anxiety Disorder** |  | 3 [6.3%]^c,d^ | 26 [26.3%]^b^ | 17 [37.0%]^b^ | 14.924 | 0.001 |
| **Eating Disorder** |  | 0 [0.0%]^d^ | 6 [6.1%] | 4 [8.5%]^b^ | 6.151 | 0.046 |
| **Affective psychosis** |  | 1 [2.1%] | 5 [5.2%] | 1 [2.1%] | 1.313 | 0.519 |

Superscript letters indicate that the group differs significantly from the other group by post hoc likelihood ratio tests at *P*<0.05. Other MINI mental disorders are not listed, as their prevalence did not differ significantly between groups at *P*>0.1.

**Figure S1. Root mean PRESS plot for the initial PLS model, showing that the optimal fit in terms of minimizing PRESS was a two-factor solution. These components maximally account for the covariation between the X and Y variables of interest. PLS is ideal for use in situations in which the X variables are correlated with each other; and when the number of variables is large relative to the number of observations.**


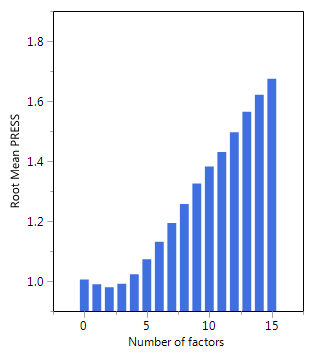


**Figure S2. Root mean PRESS plot for the final PLS model, confirming that the optimal fit in terms of minimizing PRESS was a two-factor solution.**


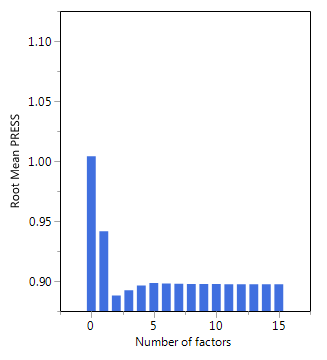


**Figure S3. Violin plots for distribution of PLS2 X scores in each group. The overall effect of group was statistically significant (F=19.881,df 3,248, P<0.001). For post hoc tests see main text. The minor component (PLS2) in the model explained 9.4% of variation in CRP, and was characterized by moderate elevation of BMI, feeling loved in childhood (high score on CTQ item 7), feeling calm (high score on STAI item 1), not wanting to change one’s family in childhood (high scores on CTQ Q10), and low scores on vegetative symptoms of depression (HAM-D items 5, 8; BDI item 15).**


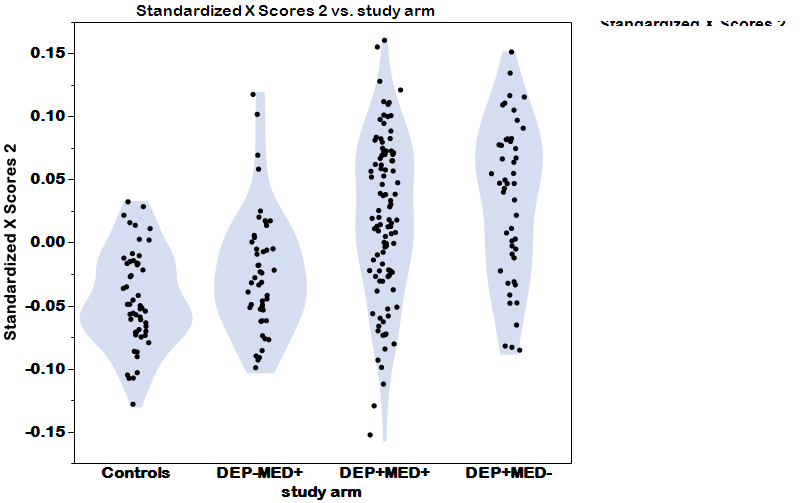


**Table S3. Sample size estimations to detect elevated BMI-corrected CRP, based on observed effect sizes.** The following table provides indications of sample sizes needed to detect a significant difference between a given MDD stratum and healthy volunteers on BMI-corrected CRP. Sample sizes were calculated using G*Power Software version 3.1.9.2, assuming alpha=0.05 two-tailed. For simplicity, 1:1 group allocation was assumed.

| Depressive Stratum | Observed effect size (Cohen’s d) versus healthy volunteers | Sample size needed to detect significant CRP difference | |
| --- | --- | --- | --- |
|  |  | 80% power | 90% power |
| Treatment-responsive | 0.29 | 188 patients, 188 healthy volunteers | 251 patients, 251 healthy volunteers |
| Treatment resistant | 0.47 | 73 patients, 73 healthy volunteers | 97 patients, 97 healthy volunteers |
| Untreated | 0.18 | 486 patients, 486 healthy volunteers | 650 patients, 650 healthy volunteers |

*References*

Beck AT, Beamesderfer A (1974). Assessment of depression: the depression inventory. *Mod Probl Pharmacopsychiatry* **7**(0): 151-169.

Bernstein DP, Fink L, Handelsman L, Foote J, Lovejoy M, Wenzel K*, et al* (1994). Initial reliability and validity of a new retrospective measure of child abuse and neglect. *Am J Psychiatry* **151**(8): 1132-1136.

Chalder T, Berelowitz G, Pawlikowska T, Watts L, Wessely S, Wright D*, et al* (1993). Development of a fatigue scale. *J Psychosom Res* **37**(2): 147-153.

Desseilles M, Witte J, Chang TE, Iovieno N, Dording CM, Ashih H*, et al* (2011). Assessing the adequacy of past antidepressant trials: a clinician's guide to the antidepressant treatment response questionnaire. *J Clin Psychiatry* **72**(8): 1152-1154.

Hamilton M (1960). A rating scale for depression. *J Neurol Neurosurg Psychiatry* **23**: 56-62.

Knesevich JW, Biggs JT, Clayton PJ, Ziegler VE (1977). Validity of the Hamilton Rating Scale for depression. *Br J Psychiatry* **131**: 49-52.

Snaith RP, Hamilton M, Morley S, Humayan A, Hargreaves D, Trigwell P (1995). A scale for the assessment of hedonic tone the Snaith-Hamilton Pleasure Scale. *Br J Psychiatry* **167**(1): 99-103.

Spielberger CD, Gorsuch RL, Lushene R, Vagg PR, Jacobs GA (1983). *Manual for the State-Trait Anxiety Inventory.* Palo Alto, CA: Consulting Psychologists Press.

Spitzer MB, Miriam G, Williams JB (1996). *Structured Clinical Interview for DSM-IV Axis I disorders, clinician version (SCID)* Washington, D.C. : American Psychiatric Press, Inc.
